# What the Heck Is Salience? How Predictive Language Processing Contributes to Sociolinguistic Perception

**DOI:** 10.3389/fpsyg.2016.01115

**Published:** 2016-08-03

**Authors:** T. Florian Jaeger, Kodi Weatherholtz

**Affiliations:** ^1^Human Language Processing Lab, Department of Brain and Cognitive Sciences, University of RochesterRochester, NY, USA; ^2^Department of Computer Science, University of RochesterRochester, NY, USA; ^3^Department of Linguistics, University of RochesterRochester, NY, USA

**Keywords:** accent, dialect, idiolect, salience, surprisal, prediction, expectation, learning

## Introduction

Some sociolinguistic variables are prone to hypercorrection, stigmatization and style shifting, while other variables are not. The status of the former type—sometimes called *stereotypes* and *markers* (Labov, 1972)—has been attributed to the increased meta-linguistic awareness language users seem to have of these variables. This awareness in turn is attributed to the *salience* of these variables, such that greater salience is assumed to cause greater meta-linguistic awareness (e.g., Trudgill, 1986). Salience has similarly been invoked when aiming to explain implicit social inferences about, or attitudes toward, speakers who exhibit certain variables in their speech (Babel, 2016; Drager and Kirtley, 2016; Squires, 2016). However, salience is a hard to define concept (for review, see Auer et al., 1998; Kerswill and Williams, 2002) and, partly as a consequence, “notoriously difficult to quantify” (Hickey, 2000). For a concept that plays such a central and ubiquitous role in sociolinguistic explanations, this is arguably a dangerous state of affairs.

This motivates the present commentary. We believe that advances in computational psycholinguistics offer definitions of sociolinguistic salience that are more concrete, both empirically and formally grounded, and quantifiable (and thus falsifiable). We propose that it is important to distinguish between the *initial salience* a listener experiences when first encountering a novel variant (e.g., because of exposure to a previously unfamiliar dialect, sociolect, or idiolect—henceforth *lects*; Schirmunski, 1930; Preston, 1996), and salience at later stages. Salience after the initial encounter is the cumulative product of an individual's experience related to the lectal variant, including direct experience, as well as discourse about the variant (e.g., explicit stereotyping or enregisterment, Agha, 2003). Here we focus on the causes for initial salience, which we think *can* be defined in a principled and quantifiable way.

Specifically, we propose that salience in the first moments when a novel lect is encountered cannot be understood without reference to prior expectations based on listeners' past language experience and the ensuing expectation violation that a listener experiences relative to those prior expectations—an idea explored in more depth by Rácz (2012, 2013). Here we contribute to these efforts. We draw on basic concepts from probability and information theory to define initial salience as a function of (top-down) prior expectations. This has several advantages. First, the proposed definition of salience is quantifiable (see also Rácz, 2013). Second, computational psycholinguistics has linked the very same quantities to language processing and learning. Recognizing this link offers the opportunity to ground sociolinguistic salience in human information processing—both empirically and theoretically—offering a parsimonious account of initial salience.

After we have outlined our proposal, we briefly turn to an apparent puzzle that was raised during the workshop leading to this special issue: several presenters pointed out that salience sometimes seems to be inversely related to the frequency of a variant and other times positively related. This puzzle readily dissolves once the view proposed here is taken into account.

## First encounters with a variant: surprisal as a measure of initial salience

Imagine a listener during the first moments of encountering a talker who speaks in an unfamiliar lect. The unfamiliar lect by definition differs from what the listener has previously experienced. Following the sociolinguistic literature, we can think of these differences as differences in the realization of linguistic variables, and the specific realization of the variables as lectal variants (Labov, 1966). What then makes a lectal variant salient in this hypothetical first encounter? Research in sociolinguistics has identified a number of perceptual features that can contribute to the perception of a variant as salient, such as a priori perceptual or articulatory distinctiveness (for review, see Auer et al., 1998). However, influences of prior experience are arguably as important or more important. Specifically, variants that are *unexpected* given the listener's *prior expectations about linguistic variables* (including, broadly speaking, the listener's language background) should be more salient in the moment they are experienced.

Events that we do not expect, or that are surprising to us, tend to stand out. There is now strong evidence that this anecdotal observation about strongly unexpected events extends to subtle and highly gradient differences in unexpectedness. During language processing, words and structures that are less expected are processed more slowly (e.g., MacDonald et al., 1994; Garnsey et al., 1997; McRae et al., 1998; McDonald and Shillcock, 2003) and they are recognized accurately less often in noise (Cole and Perfetti, 1980; Grosjean, 1980). Critically, similar costs of unexpectedness are observed for unfamiliar lectal variants when comprehenders first encounter them (e.g., Kaschak and Glenberg, 2004; Squires, 2014a; Fraundorf and Jaeger, in press). Unexpectedness—or the degree to which something is violating our expectations based on previous experience—can be measured in a number of ways. One principled measure is referred to as *surprisal* (Hale, 2001; Levy, 2008). The surprisal associated with processing a certain input (e.g., a phonetic feature, phonological category, word, or syntactic structure) is identical to the amount of new information gained by processing the input, also known as the Shannon information (Shannon, 1948).

The surprisal of a unit is defined as the logarithm of the inverse of the contextual probability of the unit:

(1) $$\begin{array}{r} I\left(unit\right)=\log\frac{1}{p\left(unit\;|\;context\right)}\;=-\log p\left(unit\;|\;context\right) \end{array}$$

If the logarithm of the inverse contextual probability is taken to base 2, surprisal measures the number of bits of information gained by processing the input over and above what was expected prior to processing the input. The surprisal of a word in (linguistic) context has been found to be proportional to its average reading time (Frank and Bod, 2011; Smith and Levy, 2013; Linzen and Jaeger, 2016). Surprisal has also been found to be correlated with neural signatures in ERP or MEG studies (Frank et al., 2015; for further references, see Kuperberg and Jaeger, 2016).

Recent studies have further linked surprisal to implicit learning operating during language processing (Fine and Jaeger, 2013; Jaeger and Snider, 2013; for a related view, see Dell and Chang, 2014). As is well-known from sociolinguistic research, talkers differ in their pronunciation, lexical, and syntactic preferences (among other things, Labov, 1972). As a consequence, efficient and robust language processing requires that linguistic expectations need to flexibly adapt to these differences (Fine et al., 2013; Kleinschmidt and Jaeger, 2015). Indeed, expectation adaptation has now been documented for speech perception (Clayards et al., 2008; for review, see Weatherholtz and Jaeger, in press), lexical (Creel et al., 2008), syntactic (Fine et al., 2013), and prosodic processing (Kurumada et al., under review), including adaptation to novel lectal variants (e.g., Kaschak and Glenberg, 2004; Bradlow and Bent, 2008; Kraljic et al., 2008; Fraundorf and Jaeger, in press). Adaptation to changes in the statistics of the environment should be sensitive to surprisal (or more generally to expectation violation): the degree to which inputs differ from prior expectations is informative about how and how much learners need to adapt their future expectations (Courville et al., 2006; Qian et al., 2012). Consistent with this prediction, there is evidence that the amount of expectation adaptation after processing unexpected linguistic input is proportional to that input's surprisal (Fine and Jaeger, 2013; Arai and Mazuka, 2014; for related evidence from production, see Bernolet and Hartsuiker, 2010; Jaeger and Snider, 2013).

Taken together, this research suggests that surprisal (or its generalization, Bayesian surprise; Itti and Baldi, 2009) is a plausible measure of “unexpectedness” and, as such, one factor that is likely to contribute to the initial salience of newly encountered lectal variants. Specifically, it is the surprisal of the variant *given the prior expectations of the listener* that is expected to predict initial salience. These prior expectations, we further submit, depend not only on *linguistic context* (e.g., the probability of a lectal variant given surrounding phonological or lexical information, including the presence or absence of other lectal variants) but also on *social context* (e.g., the probability of a lectal variant given socio-indexical information about the talker).

Consider, for example, a specific linguistic variable, such as /t/-deletion or flapping: if this variant occurs overall much more frequently in a newly encountered lect than a priori expected or in different phonological and lexical contexts than a priori expected, it will have high surprisal (this reasoning also extends to novel, not previously encountered, variables)^1^. It is in this sense that the salience of a lectal variant is *inversely* related to frequency—specifically to the *expected* relative contextual frequency of the variant^2^.

Since the expectations that determine the surprisal of a lectal variant reflect the individual's previous language experience, it naturally follows that initial salience can be “different for different social groups” (Kerswill and Williams, 2002) and individuals (see also Hickey, 2000; Campbell-Kibler, 2012). Specifically, initial salience should depend on which lects the individual has previously been exposed to, the frequency of the novel lectal variant in those familiar lect, and perhaps the frequency of similar variants in familiar lects (see Squires, 2014b). Next we turn to the question of how the initial salience of a variant is related to the probability that the variant will become associated with the lect, thereby acquiring social meaning.

## Beyond the first encounter: frequency and association

What then happens over time, as a novel lectal variant is encountered again? Consider a novel talker producing a high surprisal variant only once, compared to producing that (equally high surprisal) variant repeatedly. Intuitively, listeners should be more likely to learn an association between the variant and the novel lect in the latter case: while the surprisal of a lectal variant determines how much it “stands out,” the frequency with which the lectal variant is observed increases the probability that the variant is perceived and learned—a prerequisite to becoming associated with the lect. It is in this sense that the resulting sociolinguistic salience of a variant is *positively* related to its (*actually observed* relative) frequency *in the novel lect*. Note that this is not in conflict with our previous statement. Surprisal is predicted to cause the initial salience experienced when observing a lectal variant that was unexpected based on prior experience. High frequency in the novel lect—or specifically the cumulative effect of the surprisal experienced whenever a variant is encountered again—is predicted to increase the likelihood that the listener learns that the variant is associated with the lect (this idea is closely related to the *mutual information* between the variant and lect).

This also predicts that lectal variants can become associated almost instantaneously with a new lect or social group *if the variant is particularly unexpected* (as seems to be the case, Squires, 2014a). Such *ad-hoc* associations should be even more likely when listeners have other reasons to believe (rightly or wrongly) that the producer belongs to a novel group—a prediction that, to the best of our knowledge has not been directly tested.

Viewed this way, we can think of the sociolinguistic salience that a lectal variant acquires over time as being a function of its (perceived) informativeness about social group membership. This raises an interesting question for future research. There is now evidence that listeners develop and store implicit models or expectations about different lects that they have been exposed to (Niedzielski, 1999; Strand, 1999; Bradlow and Bent, 2008; Walker and Hay, 2011; Hanulíková et al., 2012; Shaw et al., 2015; for review, see Foulkes and Hay, 2015; Kleinschmidt and Jaeger, 2015). It is, however, still an open question to what extent the features that these implicit expectations are conditioned on are the same that more explicit processes, such as stereotyping refer to.

## Conclusion

We propose that research on sociolinguistic salience needs to take into account what is known about language processing and learning (see also Rácz, 2013; for a related perspective that grew out of the same workshop, see Schmid and Günther, 2016). One consequence of this is that the surprisal and frequency of lectal variants are likely predictors of a variant's salience. Specifically, surprisal is high when first encountering unfamiliar lectal variants. With further exposure, the association between the variant and the lect increases, while the surprisal evoked by the variant decreases.

One advantage of this approach to salience is that it makes novel testable predictions, some of which we have derived above. A second benefit is that surprisal and frequency are quantitative measures that can—in principle (provided suitable corpora)—be estimated objectively from language database. Of course, other properties of lectal variants (e.g., differences in a priori perceptual salience, such as loudness) or processes operating over them are likely to affect salience (e.g., enregisterment, which will selectively strengthen the associations between a lectal variant and the lect; Agha, 2003; Schmid, 2007). However, these other contributors to salience are generally difficult to measure reliably. We thus submit that the proposal outlined here should be taken into account *first*, providing a baseline for a variant's expected salience.

## Author contributions

All authors listed, have made equally substantial, direct and intellectual contribution to the work, and approved it for publication.

### Conflict of interest statement

The authors declare that the research was conducted in the absence of any commercial or financial relationships that could be construed as a potential conflict of interest. The reviewer DM and handling Editor declared their shared affiliation, and the handling Editor states that the process nevertheless met the standards of a fair and objective review.

## Footnotes

^1^Under the naïve assumption that everything that has never been observed is considered to have a probability of 0, the surprisal of a novel variant would be infinite. This is avoided, if some probability mass is held out to account for the fact that we do, in fact, observe novel events even as adults.

^2^There is one caveat to this prediction: prior expectations also affect *what* we perceive (cf. perceptual illusions or the perceptual magnet effect; Kuhl, 1991), and therefore can lead to a non-faithful representation of the perceptual input (cf. Feldman et al., 2009).
