# Corrigendum: What the Heck Is Salience? How Predictive Language Processing Contributes to Sociolinguistic Perception

**DOI:** 10.3389/fpsyg.2017.00902

**Published:** 2017-05-29

**Authors:** T. Florian Jaeger, Kodi Weatherholtz

**Affiliations:** ^1^Human Language Processing Lab, Department of Brain and Cognitive Sciences, University of RochesterRochester, NY, United States; ^2^Department of Computer Science, University of RochesterRochester, NY, United States; ^3^Department of Linguistics, University of RochesterRochester, NY, United States

**Keywords:** accent, dialect, idiolect, salience, surprisal, prediction, expectation, learning

In the original article, the reference for Shaw et al. (2015) was incorrectly written as:

Shaw, J. A., Catherine, T. B., Karen, E. M., Gerard, D., Bronwen, G. E., Paul, F., et al. (2015). “Effects of short-term exposure to unfamiliar regional accents: Australians' categorization of London and Yorkshire English consonants,” in *Proceedings of the 15th Australasian International Conference on Speech Science and Technology* (Christchurch), 3–5.

It should be:

Shaw, J. A., Best, C. B., Mulak, K. E., Docherty, G. J., Evans, B. G., Foulkes, P., et al. (2015). “Effects of short-term exposure to unfamiliar regional accents: Australians' categorization of London and Yorkshire English consonants,” in *Proceedings of the 15th Australasian International Conference on Speech Science and Technology* (Christchurch), 3–5.

The authors apologize for this error and state that this does not change the scientific conclusions of the article in any way.

The original article has been updated.

## Conflict of interest statement

The authors declare that the research was conducted in the absence of any commercial or financial relationships that could be construed as a potential conflict of interest.

